# Protein Interactions Network of Porcine Circovirus Type 2 Capsid With Host Proteins

**DOI:** 10.3389/fmicb.2020.01129

**Published:** 2020-06-03

**Authors:** Jianwei Zhou, Hanying Li, Tianqi Yu, Jiarong Li, Weiren Dong, Nishant Kumar Ojha, Yulan Jin, Jinyan Gu, Jiyong Zhou

**Affiliations:** ^1^MOA Key Laboratory of Animal Virology, Department of Veterinary Medicine and Center of Veterinary Medical Sciences, Zhejiang University, Hangzhou, China; ^2^Collaborative Innovation Center and State Key Laboratory for Diagnosis and Treatment of Infectious Diseases, The First Affiliated Hospital, Zhejiang University, Hangzhou, China

**Keywords:** porcine circovirus type 2, viral capsid, protein interactions network, bioinformatics approach, GO and KEGG analyses

## Abstract

Virus-host interaction is a tug of war between pathogenesis and immunity, followed by either activating the host immune defense system to eliminate virus or manipulating host immune control mechanisms to survive and facilitate virus propagation. Comprehensive knowledge of interactions between host and viral proteins might provide hints for developing novel antiviral strategies. To gain a more detailed knowledge of the interactions with porcine circovirus type 2 capsid protein, we employed a coimmunoprecipitation combined with liquid chromatography mass spectrometry (LC-MS) approach and 222 putative PCV2 Cap-interacting host proteins were identified in the infected porcine kidney (PK-15) cells. Further, a protein-protein interactions (PPIs) network was plotted, and the PCV2 Cap-interacting host proteins were potentially involved in protein binding, DNA transcription, metabolism and innate immune response based on the gene ontology annotation and Kyoto Encyclopedia of Genes and Genomes database enrichment. Verification *in vitro* assay demonstrated that eight cellular proteins, namely heterogeneous nuclear ribonucleoprotein C, nucleophosmin-1, DEAD-box RNA helicase 21, importin β3, eukaryotic translation initiation factor 4A2, snail family transcriptional repressor 2, MX dynamin like GTPase 2, and intermediate chain 1 interacted with PCV2 Cap. Thus, this work effectively provides useful protein-related information to facilitate further investigation of the underlying mechanism of PCV2 infection and pathogenesis.

## Introduction

Porcine circovirus (PCV) belongs to the genus *Circovirus* of the family *Circoviridae* (King et al., 2011). It is a small, icosahedral, non-enveloped virus and its genome is a single-stranded, closed-circular DNA (ssDNA) of 1.7 to 2.0 kb in length (Tischer et al., 1982; King et al., 2011). There are four genotypes of PCV: PCV1, PCV2, PCV3, and PCV4. PCV1, a non-pathogenic virus, was first detected as a contaminating agent in porcine kidney (PK-15) cell lines (Tischer et al., 1974, 1982). PCV2 is the major causative agent of porcine circovirus-associated diseases (PCVAD) inducing a variety of progressive disease syndromes, including postweaning multisystemic wasting syndrome (PMWS), porcine dermatitis and nephropathy syndrome (PDNS), porcine respiratory disease complex (PRDC), and porcine reproductive disorders (Thomson et al., 2000; Opriessnig et al., 2007; Rodriguez-Carino and Segales, 2009). Recently, PCV3 was identified and shown to be associated with porcine dermatitis, reproductive failure, and nephropathy syndrome (Phan et al., 2016; Palinski et al., 2017; Jiang et al., 2019). PCV4 is a newly identified virus and considered as a distinct circovirus species and associated with severe clinical disease involving respiratory system, gastrointestinal system, and PDNS (Zhang et al., 2019). Therefore, PCV undoubtedly causes enormous loss to pig industry worldwide as well as to the global economy.

The genome of PCV contains 11 potential opening reading frames (ORFs) (Hamel et al., 1998; Cheung, 2003; Zhou et al., 2006). Among these, only five viral proteins have been identified and characterized so far. Namely, the ORF1-encoded Rep and Rep’, collectively known as replicase proteins, are necessary for the rolling-circle replication of PCV genomic DNA (Mankertz et al., 1998; Mankertz and Hillenbrand, 2001; Cheung, 2006). The ORF2-encoded capsid protein (Cap) is necessary for virion packaging and may participate in genome replication by interacting with Rep protein and is also the main viral immunogen and acts as a key regulator of viral replication as well as the virus-host interaction (Nawagitgul et al., 2000; Blanchard et al., 2003; Fenaux et al., 2004; Timmusk et al., 2006; Wiederkehr et al., 2009; Cao et al., 2015; Fermin and Tennant, 2018; Wang H. J. et al., 2019). The remaining three identified viral proteins are involved in the biological processes of PCV2 infection but not in PCV2 replication (Liu et al., 2005; He et al., 2013; Lv et al., 2015; Lin et al., 2018).

Porcine circovirus 2 (PCV2) is one of the smallest DNA virus of animals. Its genome encodes only a small set of proteins and PCV2 relies highly on host cellular machinery for its multiplication. There are previous reports about the interactions between host proteins and PCV capsid protein using bacterial or yeast two-hybrid or coimmunoprecipitation assays (Timmusk et al., 2006; Finsterbusch et al., 2009; Zhang et al., 2009; Liu et al., 2013; Cao et al., 2015; Wang Z. J. et al., 2018; Wang T. T. et al., 2019). Constant interactions between the virus and host during the process of co-evolution have reshaped the antiviral innate immune system of the host and, in turn, the capability of viruses to manipulate host defend system to facilitate their propagation (Stebbing et al., 2003). The general approaches to screen interacting-host proteins proved to be insufficient to study the detailed mechanisms of the replication and pathogenesis of PCV2. However, newly developed high throughput proteomic techniques have proved to be useful in identifying large set of interacting partners in case of other viruses but has not been utilized for PCV2.

Hence, in the present study, in order to gain a more detailed knowledge of the virus-host interaction, we employed a coimmunoprecipitation coupled with liquid chromatography mass spectrometry (LC-MS) approach and identified 222 putative host proteins interacting with PCV2 capsid protein in the infected PK-15 cells and then constructed a protein-protein interactions (PPIs) network. We also performed bioinformatic analyses of the interactions network. The gene ontology annotation and pathway enrichment analyses showed that the PCV2 Cap-interacting host proteins belong to different cellular pathways such as protein binding, DNA transcription, metabolism and innate immune response, including the mitogen-activated protein kinase signaling and spliceosome pathway. We then demonstrated that PCV2 Cap interacted *in vitro* with hnRNPC, NPM1, DDX21, IPO5, EIF4A2, SNAI2, MX2, and IC1, although they displayed different binding capacity. Thus, the results of the current study will be useful in identifying novel antiviral therapeutic targets against PCV2 infection.

## Results

### Identification of Cap-Host Protein Interactions in the PCV2 Infected PK-15 Cells by Liquid Chromatography Mass Spectrometry

**FIGURE 1:**
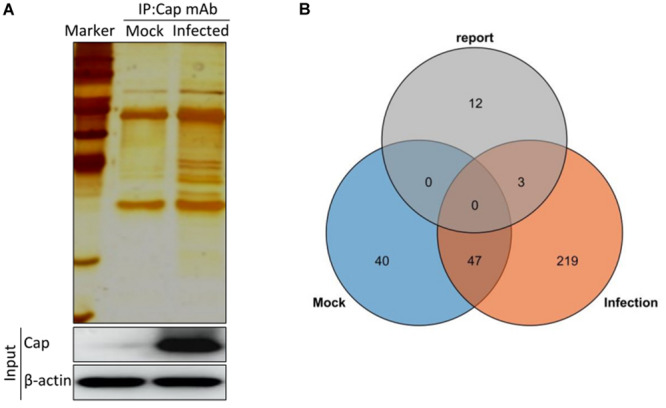
Characterization of PCV2 Cap-interacting cellular proteins. **(A)** Mock infected PK-15 cells or infected with PCV2 at MOI of 1.0 were harvested 48 hpi and coimmunoprecipitation assay was performed by purified anti-Cap IgG. PCV2 Cap-interacting host proteins were eluted with protein A/G sepharose and analyzed on SDS-PAGE followed by silver staining. β-actin was used as a internal loading control. For **(A)**, lane 1, protein molecular weight ladder; lane 2, Mock-infected; lane 3, PCV2-infected. **(B)** Venn diagram of the identified protein candidates interacting with PCV2 Cap from mock infected, PCV2 infected and reported previously, respectively. Blue, orange, and gray colors indicate proteins from mock infected, PCV2 infected and reported previously, respectively. Common proteins within the data sets have been indicated in the colored intersections. Proteins have been represented as the respective NCBI gene names (Supplementary Table S1).

To identify the potential host proteins binding to PCV2 Cap in the infected PK-15 cells, we performed coimmunoprecipitation assays combined with LC-MS in PK-15 cells with or without PCV2 infection. The cell lysates were immunoprecipitated using the purified anti-Cap IgG at 48 hpi and subjected to silver staining for visualization of the cellular proteins bound to PCV2 Cap (Figure 1A). As a negative control, mock infected PK-15 cell lysates was used to eliminate the non-specific interactions. Distinct bands could be observed as compared to the negative control. Moreover, LC-MS is further employed to unravel the Cap interactions with the host proteome. A total of 269 PCV2 Cap-interacting cellular proteins were identified and a list of partial unique host proteins was shown in Table 2 and Supplementary Table S1. Among them, 47 proteins were also present in mock cell lysate and hence were not considered for further interpretation and remaining, 222 differentially expressed cellular proteins were considered as novel cellular candidates interacting with PCV2 Cap in the infected PK-15 cells (Supplementary Table S2). We also performed literature search about the PCV2 Cap-interacting proteins and compiled the information about 15 proteins reported in previous papers (Timmusk et al., 2006; Finsterbusch et al., 2009; Zhang et al., 2009; Liu et al., 2013; Cao et al., 2015; Wang Z. J. et al., 2018; Wang T. T. et al., 2019). Among those 15, our experiment identified 3 proteins namely NPM1, heat shock protein 70, and IC1 (Figure 1B).

### Construction and Analysis of the Protein-Protein Interactions Network

**FIGURE 2:**
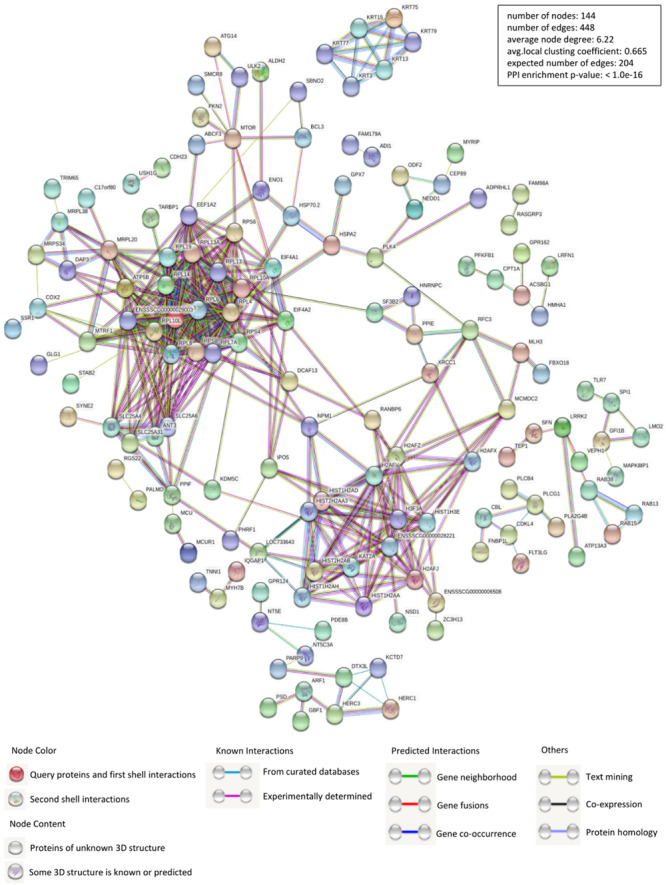
Construction and analysis of the protein-protein interactions (PPIs) network using STRING database. Each edge color indicates a different method of PPIs prediction as indicated below the figure. The map of PCV2 Cap-interacting host proteins further interacting with the other proteins of our data were constructed and plotted by using the network analyzer tool of “Cytoscape version 3.7.1.” The corresponding symbols indicating different protein classes have been mentioned on the figure. Proteins have been represented as the respective NCBI gene names (Supplementary Table S2).

Identification of PPIs is at the center of molecular biology considering the unquestionable role of proteins in cells. Accordingly, we constructed the interactions network of PCV2 Cap interacting host proteins-cellular proteins using STRING database and applied for network structure as well as function relationship study (Figure 2 and Supplementary Table S2). Along with the experimentally confirmed interactions, those predicted from gene fusion, co-expression, homology and text mining were also considered for network construction and analysis. The cellular proteins of the interactions network were mainly clustered into the categories of ribosome biogenesis regulation, metabolism and cytoskeletal rearrangement. The observed number of edges for the network (448) was significantly higher than the expected (204) for the given number of nodes (144), implying that there are more interactions than expected and the proteins from our data exhibited more interactions than expected for a random set of proteins. Such enrichment indicated that the proteins are partially clustered as a group of having role mainly during replication/transcription.

### Gene Ontology Annotation and Analysis

**FIGURE 3:**
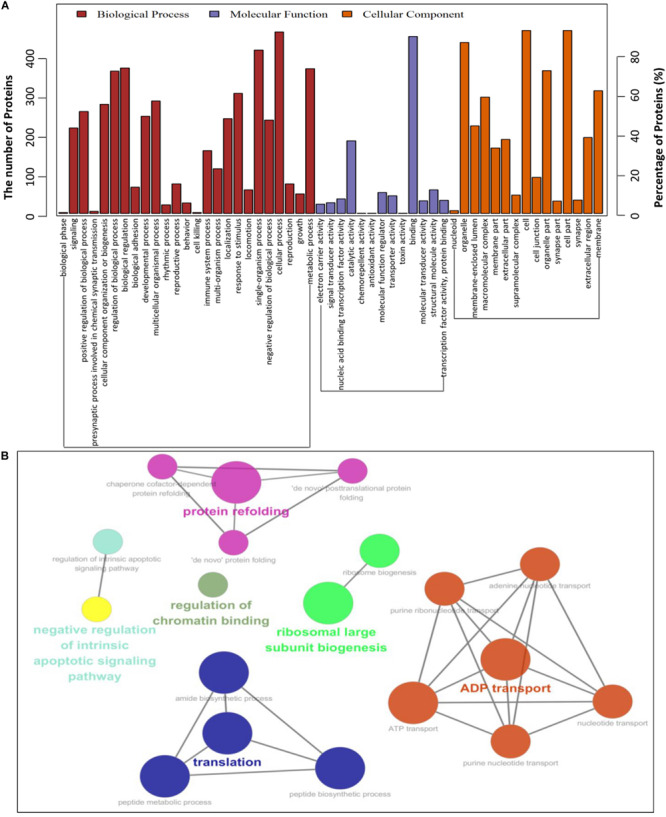
Gene ontology analysis of identified PCV2 Cap-host interactome. **(A,B)** Representative overrepresented GO terms of protein clusters and the GO distribution of all number of proteins in the PCV2-infected cells versus mock-infected were classified into three categories using software Cytoscape 3.7.1 with plugin GOclue and were shown for significantly enriched terms based on biological process (BP), molecular function (MF), cellular component (CC), *p*-value < 0.05. The Roman numerals represented the detailed GO terms as shown in Supplementary Tables S3, S5.

To establish the host cell proteins or pathways that have been enriched in the PCV2 Cap-cellular proteins interactions network, we performed gene ontology annotation and analysis of the target proteins differentially expressed in the PCV2 infected PK-15 cells (Supplementary Table S2) to predict the biological function. GO annotation and analysis were done for three categories: biological process, molecular function and cellular component. Apparently, a large number of biological processes, like ribosomal biogenesis, regulation of metabolic process, protein folding, protein transport and translation, and ADP transport were enriched. Furthermore, protein binding, regulation of catalytic activity, signal transducer and transporter were enriched under the category of molecular function and membrane-enclosed lumen, extracellular region and cell organelles were enriched under the category of cellular component (Figures 3A,B and Supplementary Tables S3, S5). Collectively, the GO annotation and analysis of target proteins inferred that the PCV2 Cap may perturb several processes such as protein binding, DNA replication and transcription, metabolism, and innate immune response.

### KEGG Pathway Enrichment Analysis

**FIGURE 4:**
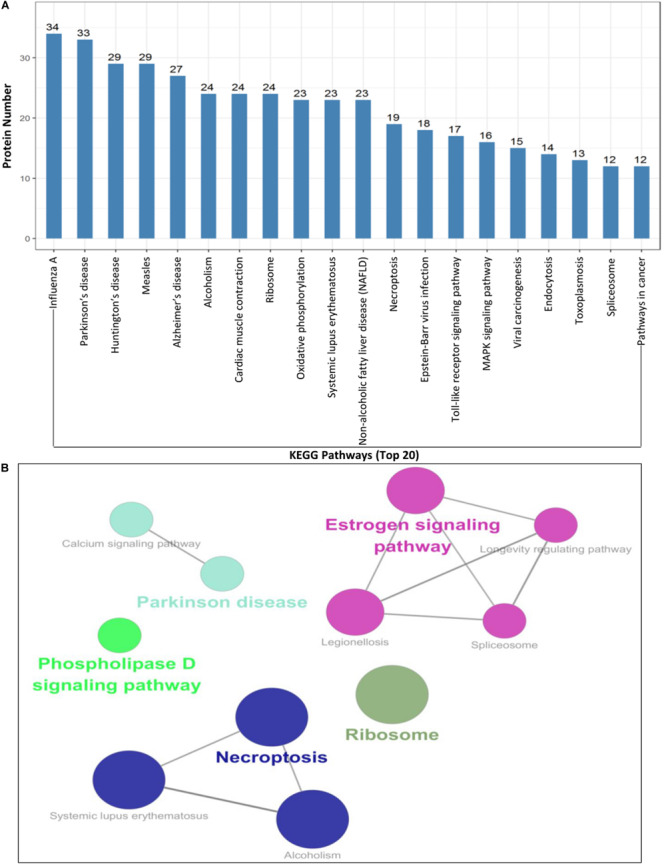
KEGG pathway enrichment analysis. **(A,B)** Graph shows the enriched pathways targeted by PCV2 Cap-interacting proteins, analyzed by KEGG functional annotation pathway database (Supplementary Tables S4, S5) using software Cytoscape 3.7.1 with plugin GOclue (Supplementary Figures S1A,B). Schematic representation of the MAPK signaling pathway (imported from KEGG: map04010) and the spliceosome pathway targeted by PCV2 Cap-interacting proteins (imported from KEGG: map03040). Proteins interacting with PCV2 Cap within the entire pathway are depicted red color.

To further understand and predict the cellular pathways of metabolism and signal transduction of Cap-interacting host protein candidates targeted by PCV2 (Supplementary Table S2), we conducted pathway enrichment analysis using the KEGG and the top 20 enriched pathways with the highest representation of each term were enlisted. Interestingly, the majority of the target proteins were involved in influenza A, Parkinson’s disease, Estrogen signaling pathway, necroptosis, toll-like receptor signaling pathway and so on. The KEGG enrichment analysis suggested that pathways involved in immune response, regulation of necroptosis and endocytosis were preferentially targeted (Figures 4A,B and Supplementary Tables S4, S5). Furthermore, enrichment analysis also indicated that the proteins might participate in the regulation of the expression of other proteins of different processes such as ribosomal biogenesis, phospholipase D signaling pathway and MAPK signaling pathway. We have shown the schematic of the entire pathway networks for MAPK signaling and spliceosome from the KEGG database (Supplementary Figure S1).

### Validation of Interactions Between Cellular Proteins With PCV2 Capsid Protein

**FIGURE 5:**
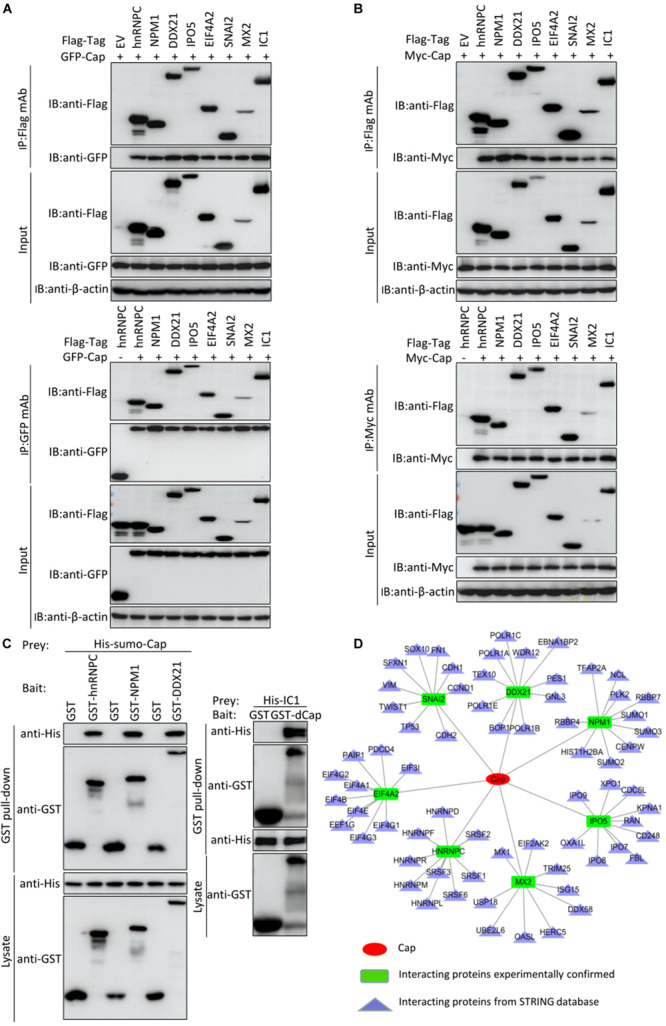
Validation of Cap-host protein interactions. **(A,B)** HEK-293T cells were co-transfected with plasmids expressing FLAG-hnRNPC, FLAG-NPM1, FLAG-DDX21, FLAG-IPO5, FLAG-EIF4A2, FLAG-SNAI2, FLAG-MX2, or FLAG-IC1 and plasmids expressing GFP-Cap **(A)**, Myc-Cap **(B)**, respectively. Among them, GFP-Cap **(A)**, Myc-Cap **(B)**, FLAG-hnRNPC **(A,B)** co-transfected with empty vector were served as negative controls and GFP-Cap **(A)**, Myc-Cap **(B)** co-transfected with FLAG-IC1 were served as positive controls, respectively. The cell lysates were immunoprecipitated with FLAG beads, anti-GFP, or anti-Myc mAbs, separated by SDS-PAGE, western-blotted and detected with corresponding antibodies, respectively. β-actin was served as internal loading control. **(C)** Purified His-sumo-Cap was separately added to the GST, GST-hnRNPC, GST-NPM1, GST-DDX21 proteins and His-IC1 was also separately added to the GST, GST-dCap proteins and then subjected to GST pull-down assays and immunoblotted with corresponding antibodies, respectively. **(D)** PCV2 Cap-host interactions network. Interaction map of PCV2 Cap with interacting host proteins was constructed by software Cytoscape 3.7.1. Proteins were classified on the basis of their protein class. The corresponding symbols indicating different protein classes have been mentioned on the figure (Supplementary Table S6).

To further validate the protein interactions obtained from mass spectrometry, we conducted *in vitro* coimmunoprecipitation assays. Eight cellular proteins from the PCV2 infected test samples were randomly selected to confirm the mass spectrometry data. 293T cells were cotransfected with GFP-Cap and empty vector, FLAG-hnRNPC, NPM1, DDX21, IPO5, EIF4A2, SNAI2, MX2, IC1, and then subjected to immunoprecipitation with Flag beads or anti-GFP purified monoclonal antibody (mAb). Results showed that PCV2 Cap interacted specifically with hnRNPC, NPM1, DDX21, IPO5, EIF4A2, SNAI2, MX2, and IC1, and no signal was observed with empty construct, although they showed different binding capacity (Figure 5A). In order to eliminate the role of GFP tag on the authenticity of the results, we performed similar assay with Myc-Cap and the results were consistent with those of the GFP-Cap plasmid (Figure 5B). To investigate whether Cap bound directly to the indicated cellular proteins, we performed glutathione S-transferase pull-down experiments. His-sumo-Cap together with GST, GST-hnRNPC, GST-NPM1, GST-DDX21, and His-IC1 together with GST, GST-dCap were respectively subjected to GST pull-down assays and immunoblotting. As shown in Figure 5C, the products of GST-hnRNPC, GST-NPM1, GST-DDX21 could pull down His-sumo-Cap and GST-dCap could also pull down His-IC1, but no pull-down was observed when empty vector was used. Taken together, these data indicated that PCV2 Cap interacted directly with hnRNPC, NPM1, DDX21, and IC1. The results obtained from the coimmunoprecipitation and glutathione S-transferase pull-down experiments validate the data generated from the LC-MS based proteomic analysis. Then, we constructed another interactions network of the experimentally confirmed host protein-PCV2 Cap interaction and the cellular partners of the Cap interacting host proteins *in silico* using Cytoscape 3.7.1 software (Figure 5D and Supplementary Table S6) and it might contribute to investigate the potential role of PCV2 Cap in the viral lifecycle as well as the replication and pathogenesis of PCV2.

## Discussion

Porcine circovirus type 2 (PCV2) is one of the most common viruses which infects pigs globally. It is the primary etiological factor of PCVAD, which is responsible for massive loss for the swine production worldwide as well as to the global economy (Opriessnig et al., 2007). PCV2 could function as a model for the study of virus life cycle, however, the molecular mechanism of PCV2 replication and pathogenesis, as well as the dependency of viral propagation on host factors are still poorly understood. Actually, the virus-host interaction plays the crucial role in the virus life cycle, and it also initiates the battle between pathogenesis and host immunity. The capsid protein of circovirus is a critical regulator of genome replication together with replicase protein and is essential for virus multiplication (Timmusk et al., 2006; Finsterbusch et al., 2009). Thus, it is hypothesized that PCV2 capsid and replicase proteins may form replicase complex by interaction with a set of host proteins and the multiprotein complex perhaps has a role during various stages of virus life cycle such as uncoating, assembly, nuclear entry and egress. It is currently unknown whether PCV2 capsid protein (Cap) has any direct role in subverting or utilizing the host cellular machinery for its own benefit. Additionally, the functional domains of capsid protein responsible for the binary interaction with host proteins are also unknown.

There are several reports about the interactions of PCV capsid and host proteins (Timmusk et al., 2006; Finsterbusch et al., 2009; Zhang et al., 2009; Liu et al., 2013; Cao et al., 2015; Wang Z. J. et al., 2018; Wang T. T. et al., 2019). In this study, we have screened and identified 269 putative cellular proteins interacting with PCV2 capsid protein and 222 new host proteins were specific to the PCV2 infected PK-15 cells compared to the mock infected using a coimmunoprecipitation coupled with LC-MS at an MOI of 1.0 for 48 h (Figure 1), which belongs to the qualitative analysis proteomics. Among them, although the specific interaction host proteins cannot show the fold changes of the expression levels under PCV2 infection, they can be served as the candidates for further research. However, in our previous paper (Zhou N. et al., 2017), we have used isobaric tags for relative and absolute quantitation (iTRAQ)-coupled LC-MS/MS to identify the differential proteome within PCV2-CSFV coinfection at an MOI of 10.0 for 64 h, which belongs to the quantitative analysis proteomics. Among them, there are as many as 788 differential proteins identified, which can exhibit the fold changes of the expression levels of different host proteins under PCV2-CSFV coinfection. Besides, both all utilized the LC-MS to screen differential proteins. All in all, for the ongoing study, we advise the researchers to combine the both methods to select the target proteins.

Subsequently, a PPIs network was plotted (Figure 2). The Gene ontology (GO) and Kyoto Encyclopedia of Genes and Genomes (KEGG) pathway analyses indicated that the interacting cellular proteins participate in different cellular processes such as protein binding, DNA transcription and splicing, metabolism and innate immune response, including MAPK signaling pathway and spliceosome pathway (Figures 3, 4). We also validated the binary interactions of PCV2 Cap with selected host proteins such as hnRNPC, NPM1, DDX21, IPO5, EIF4A2, SNAI2, MX2, IC1 *in vitro* by coimmunoprecipitation and GST pull-down assays (Figure 5). These interactions information of Cap and cellular proteins in PCV2 infected cells provided a potential insight for studying PCV2 replication and pathogenesis. Our current study is the first of its kind which utilized the modern proteomic tools such as liquid chromatography mass spectroscopy to identify the cellular partners of PCV2 capsid protein. Our data constitutes a repertoire of cellular proteins which will be useful for further study of PCV replication and pathogenesis. The bioinformatic analyses of available dataset will be useful for better understating of the role of host proteins in the cellular pathways. Advances in tools for data acquisition, processing, integration and computation could provide rapid, and precise strategies for the development of novel therapies for infectious diseases (Peng et al., 2009; Aderem et al., 2011; Xue and Miller-Jensen, 2012). Therefore, we hypothesized that the set of host proteins interacting with PCV2 capsid protein may form a differential replicase complex which probably play crucial roles in the replication and pathogenesis of PCV2.

Certain cellular processes such as protein binding, DNA transcription, metabolism and innate immune response, including MAPK signaling pathway and spliceosome pathway are important during PCV2 infection and hence need special attentions during further studies. Although, in the present study we have identified several proteins of these pathways, the exact roles of them are still unknown during the life cycle of PCV2 and further study is necessary in that regard. An important characteristic of virus-host interaction is the manipulation of innate immune response in order to establish favorable cellular environment for efficient viral propagation. It is hypothesized that PCV2 might hijack the innate immune response and cellular splicing pathways. To support this hypothesis, our data of GO annotation and KEGG enrichment analyses show that there is an enrichment of these pathways-related proteins (Figures 3, 4). In the present study, some proteins of innate immune response pathways, such as interferon regulatory factor 7 (IRF7), interferon-stimulated gene 54 (ISG54), DDX21, Janus kinase 1 (JAK1), MX2, toll like receptor 7 (TLR7) were found to interact with PCV2 Cap and there is a need of further study to better understand the host innate immune response against PCV infection.

Proteins belonging to the heat shock protein (HSP) family have roles as chaperons in protein folding. These proteins play similar roles in the maintenance of proper folding and stabilization of viral proteins (Seo et al., 2018). During viral infections, host cell undergoes stress and the cellular stress results in unfolded protein response (UPR) (Geller et al., 2012; Wang R. et al., 2018). In our study, we found enrichment of certain proteins, such as HspA1A, HspA1B, and HspA6 which have roles in UPR at endoplasmic reticulum and the UPR at host ER machinery is reported to be relevant to PCV2 infection (Zhou et al., 2016; Zhou Y.S. et al., 2017; Ouyang et al., 2019). Our study also shows the enrichment of the spliceosome pathway. Different heterogeneous nuclear ribonucleoproteins, such as hnRNPC, and hnRNPU were identified to interact with PCV2 Cap (Figure 5). Spliceosomal complex proteins help in the generation of stable RNA structures, and ribonucleoproteins play roles in the stability and transport of the RNA (Will and Luhrmann, 2011). Previous studies have reported that hnRNPC protein was involved in the infection of hepatitis delta virus (HDV), dengue virus (DENV), Japanese encephalitis virus (JEV), and hnRNPU protein functioned as a nuclear sensor for viral RNA (Casaca et al., 2011; Dechtawewat et al., 2015; Mukherjee et al., 2017; Cao et al., 2019).

In the present study, the Cap-host protein interactions were systematically screened for the first time in the PCV2 infected PK-15 cells. The PPIs network was constructed and the potential functions of identified host proteins were predicted by GO and KEGG enrichment analyses. All of the randomly selected eight proteins could bind to PCV2 Cap in the Co-IP and GST pull-down assays. The results of the interactions of PCV2 Cap with host proteins and the interpretation of the virus-host interactions network might be helpful to better understand the putative mechanisms or pathways through which PCV2 exerts the pathogenic effects. Moreover, the knowledge about the host proteins targeted and the pathways perturbed by PCV2 Cap might contribute to a comprehensive understanding of virus-host interaction and provide new insights into the identification of novel targets for both host effectors and virus factors, and ultimately be useful in designing better therapeutic strategies against the PMWS and other PCVAD. Our data also suggest that the replication mechanism and pathogenesis of PCV2 into the host are a complex phenomenon which require further study.

## Materials and Methods

### Cells and Virus

Immortal porcine kidney epithelial PK-15 cells (ATCC-CCL33, American Type Culture Collection), which were free of PCV and maintained in our laboratory, were routinely cultured at 37°C and 5% CO2 in minimal essential medium (MEM; Gibco, Carlsbad, CA, United States). HEK293T cells (CRL-11268; ATCC) were cultured at 37°C and 5% CO2 in Dulbecco’s modified Eagle medium (DMEM; Gibco). All media were supplemented with 10% fetal bovine serum (CCS30010.02; MRC, Australia) as previously described (Lin et al., 2018). PCV2 strain HZ0201 (accession no. AY188355.1), stored in our laboratory, was originally isolated from pig herds with naturally occurring PMWS and propagated in PK-15 cells (Zhou et al., 2006). The PK-15 cells were infected with PCV2 at the MOI of 1.0 for 48 h in the figures, figure legends, or text.

### Antibodies and Reagents

Rabbit polyclonal antibody (pAb) against Myc (R1208-1), GFP (SR48-02), FLAG (0912-1), and mouse monoclonal antibody (mAb) against β-actin (M1210-2), GST (M0807-1) were purchased from Huaan Biological Technology (Hangzhou, China). Mouse anti-Myc (05-419) and anti-FLAG (F1804) mAbs were both obtained from Sigma-Aldrich (United States). Anti-FLAG affinity resin (A2220) for immunoprecipitation was also purchased from Sigma-Aldrich. Mouse monoclonal antibody (mAb) against GFP (B-2, sc-9996) was purchased from Santa Cruz Biotechnology. Glutathione agarose beads for GST pull-down were purchased from Thermo Scientific (United States). Mouse mAb to Cap of PCV2 was produced by our laboratory (Zhou et al., 2005). Cell NP-40 lysis buffer [50 mM Tris (pH 7.4), 150 mM NaCl, 1% NP-40] was purchased from Beyotime (P0013F; Shanghai, China). Horseradish peroxidase (HRP)-labeled goat anti-mouse and anti-rabbit IgG were purchased from KPL (Milford, MA, United States).

### Plasmid Construction and Cell Transfection

The full-length capsid open reading frame (ORF) from PCV2 HZ0201 genome was amplified by PCR and subcloned into the vectors pCMV-Myc-N (Clontech, Palo Alto, CA, United States), pEGFP-C3 (Clontech). His-sumo-Cap was synthesized and inserted into the vector pET-28a (Novagen, Madison, WI, United States) by Sunya Biotechnology (Zhejiang, China). FLAG-hnRNPC, FLAG-NPM1, FLAG-DDX21, FLAG-IPO5, FLAG-EIF4A2, FLAG-SNAI2, FLAG-MX2 expression plasmids and GST-hnRNPC, GST-NPM1, GST-DDX21 plasmids were constructed by respectively inserting the corresponding ORFs into the pCMV-Flag-N (631604; Clontech) and pGEX-4T-1 (GE Healthcare Biosciences, Piscataway, NJ, United States) vectors as indicated. GST-dCap, FLAG-IC1, and His-IC1 plasmids were constructed and stored by our laboratory (Cao et al., 2015). The full-length cDNA sequences of indicated genes were amplified from PK-15 cells by use of specific primers. The primers used are summarized in Table 1. For transfection, 293T cells was seeded onto the designated plates at a suitable density according to the experimental schemes and grown to 70–90% confluence. All constructed DNAs were transfected into cells using the ExFect transfection reagent (T101-01/02; Vazyme Biotechnology, Nanjing, China) according to the manufacturer’s instructions.

**TABLE 1:** List of primers used for cloning in the study.

**Gene product**	**Sense primer (5′ to 3′)**	**Antisense primer (5′ to 3′)**
Cap	ATGACGTATCCAAGG AGGC	TTAAGGGTTAAGTGGGG GGTCT
hnRNPC	ATGGCCAGCAACGTTACC AACAAGA	CTAAACCCCACTATGTG CTTGAGAG
NPM1	ATGGAAGATTCGATG GATAT	TTAAAGAGACTTCCT CCACT
DDX21	ATGCCGGGGAAACTT CGTAGT	TTACTGTCCAAACGC TTTGCT
IPO5	ATGGCGGCGGCCGCGG CGGAGCAGC	TCAAGCAGAGTTCAGGA GCTCCTGT
EIF4A2	ATGTCTGGTGGCTCC GCGGAT	TTAAATAAGGTCA GCCACATT
SNAI2	ATGCCGCGCTCCTT CCTGGT	TCAGTGTGCCACAC AGCAGC
MX2	ATGCCTAAACCCCGCA TGTCGT	TTACATCCCTTGT ACCTCAAC

### SDS-PAGE and Immunoblotting

For western blotting, the beads were lysed in lysis buffer after Co-IP or GST pull-down assays, and proteins were separated by standard SDS-PAGE and transferred to nitrocellulose membranes (GE Healthcare) followed by blocking in PBS containing 5% skim milk and 0.05% Tween 20 and washing with PBS containing 0.05% Tween 20. The membranes were incubated with primary antibody overnight at 4°C. After three to five washes with PBS containing 0.1% Tween 20, the membranes were incubated with HRP-labeled secondary antibody at room temperature for 1.0 h. Immunoreactive protein bands were then visualized using enhanced chemiluminescence (Amersham Biosciences, Little Chalfont, United Kingdom) and imaged using AI680 Images (GE Healthcare).

### Co-IP and GST Pull-Down Assays

To identify interacting host proteins with PCV2 Cap during virus infection, PK-15 cells were infected with PCV2 at an MOI of 1.0 for 48 h and the resultant cells were harvested and lysed with NP-40 buffer containing PMSF for 1.0 h at 4°C. After centrifugation at 12,000 × *g* for 10 min, the supernatant was treated with protein A/G plus agarose (Santa Cruz Biotechnology, Santa Cruz, CA, United States) for 1.0 h at 4°C and then incubated with the purified anti-Cap IgG at 4°C overnight. Protein A/G plus agarose was then added to the immune complexes and incubated for 4.0 h at 4°C. Finally, the beads were washed three to five times with lysis buffer and boiled with sample buffer for 10 min. The samples were then subjected to 12% SDS-PAGE followed by silver staining. β-actin was used as a internal loading control. To detect interactions between PCV2 Cap and hnRNPC, NPM1, DDX21, IPO5, EIF4A2, SNAI2, MX2, or IC1, HEK293T cells were transfected with the indicated plasmids for 48 h. Cells were lysed in NP-40 buffer containing a protease inhibitor cocktail. After centrifugation at 12,000 × *g* for 10 min, the supernatant was treated with protein A/G plus agarose (Santa Cruz Biotechnology, Santa Cruz, CA, United States) for 1.0 h at 4°C to eliminate non-specific binding to the agarose gel, and immunoprecipitated using anti-FLAG beads, anti-GFP, or anti-Myc mAbs. The beads were washed three times with NP-40 buffer and then boiled in protein loading buffer before the proteins were separated and subjected to western blotting. For the GST pull-down assays, His-sumo-Cap, His-IC1, GST, GST-hnRNPC, GST-NPM1, GST-DDX21, and GST-dCap were expressed in *Escherichia coli* BL21 cells. His-sumo-Cap and His-IC1 were purified using Ni-NTA agarose (30210; QIAGEN, Germany). GST, GST-hnRNPC, GST-NPM1, GST-DDX21, and GST-dCap were purified using Pierce glutathione agarose (21516; Thermo, Rockford, IL, United States). To prepare the bait proteins, purified GST, GST-fusion proteins were immobilized on glutathione agarose beads, while His-fusion proteins were used as the prey protein. A total of 500 ng of His-sumo-Cap was respectively added to the GST, GST-hnRNPC, GST-NPM1, and GST-DDX21 proteins and a total of 500 ng of His-IC1 was also respectively added to the GST, GST-dCap proteins and incubated overnight at 4°C and then washed with NP-40 lysis buffer and boiled with sample loading buffer. Finally, the samples were subjected to 12% SDS-PAGE and immunoblotted with anti-His mAb (our unpublished data) and anti-GST mAb.

### Liquid Chromatography Mass Spectrometry (LC-MS)

The silver staining gels from coimmunoprecipitation experiments were mixed together and subjected to protein identification by LC-MS analysis in APTBio (Shanghai, China). Peptides were concentrated and desalted on an EASY column (2 cm × 100 μm 5 μm-C18; 75 μm × 100 mm 3 μm-C18; Thermo Finnigan, San Jose, CA, United States) and online eluted on an analytical RP column (0.18 150 mm BioBasic-18, Thermo Electron). For that, a 60-min gradient from 4 to 50% B (solvent A: 0.1% v/v formic acid; solvent B: 0.1% v/v formic acid, 84% v/v ACN) 0 min–50 min, from 50 to 100% B 50 min–54 min, 100% B 54 min–60 min, were used. Protein searches were carried out using Mascot 2.2 software. Protein identification was performed with the following criteria: (a) Trypsin digested peptides with 2 max missed cleavages allowed, (b) Proteomics Tools: 3.1.6, (c) >1 unique peptides, (d) Filter by score ≥20. Fasta files for pig Proteome database (35484) downloaded (20161206) from the UniProt were used for protein searches. Proteins found in respective negative control sample were eliminated from the dataset to remove non-specific interactions. Proteins represented by at least two unique peptides were considered for further analysis and the proteins in the negative control were excluded.

### Construction and Analysis of the Protein-Protein Interactions Network

All the experimentally derived data sets were used to generate PCV2 Cap-host proteins interactions network by using “Cytoscape version 3.7.1.” To analyze the interactions among host proteins, STRING database was used. Only interactions confirmed by direct physical binding were considered for plotting inter-protein interaction map. Topological parameters and central measures of the network were calculated by using a network analyzer tool in Cytoscape 3.7.1. Pig protein-protein interaction analysis was also performed by using STRING database. In the all networks and throughout the study, we have used NCBI gene names to represent the proteins in order to have a consensus in protein accession. Corresponding NCBI gene names have been listed separately (Supplementary Table S2). The list of partial unique proteins identified to be interacting with PCV2 Cap is shown in Table 2.

**TABLE 2:** List of partial unique PCV2 Cap-interacting host proteins.

**Gene symbols of partial unique interacting proteins**
EIF4A2, RPL4, EIF4A1, ENO1, PSMD3, CPT1A, KRT3, MAPK8IP1, ULK2, EIF5A, ATP5B, COX2, PLK4, MYH7B, ADI1, FUBP1, SNAI2, HNRNPC, ARF3, TRIM7, NCOR2, KAT2A, TLR7, HSPA1L, RANBP6, ISG54, JAK1, IRF7, TLR7, EEF1A1, MLH3, TARBP1, RBBP6, CBL, NEDD1, TCEA1, HSPA1B, HSPA1A, KDM5A, MDA5, IPO5, RPS8, KRT79, ANXA11, KCTD7, EEF1A2, RAB38, CAMSAP1, ZC3H13, TMEM237, KDM5C, ALDH2, LRRTM4, TRIM65, DTX3L, DDX21, MYRIP, ITIH2, CBL, MTOR, USH1G, TNIP2, RAB15, IC1, NPM1, HSPA6

*Proteins have been represented as the respective NCBI gene names.*

### GO and KEGG Pathway Analyses

Gene ontology (GO) analysis was applied using software Cytoscape 3.7.1 with plugin GOclue to annotate the genes with terms of cellular component (CC), biological process (BP), and molecular function (MF) based on the GO database (Supplementary Tables S2, S3, S5). Kyoto Encyclopedia of Genes and Genomes (KEGG) enrichment was conducted to predict the mitogen-activated protein kinase signaling and spliceosome pathway based on the KEGG database (Supplementary Tables S2, S4, S5). The pathways of GO and KEGG with the corrected *p*-value < 0.05 were chosen to be significantly enriched, respectively. GO and KEGG pathway analyses were completed by APTBio (Shanghai, China).

## Data Availability Statement

All datasets generated for this study are included in the article/Supplementary Material.

## Author Contributions

JwZ, HL, and TY conceived and designed the experiments. JwZ, YJ, and JG designed and performed the experiments. JwZ, JL, WD, and NO analyzed the data. JwZ and JyZ wrote, reviewed, revised, and edited the manuscript. All authors have read and agreed to the published version of the manuscript.

## Conflict of Interest

The authors declare that the research was conducted in the absence of any commercial or financial relationships that could be construed as a potential conflict of interest.

## Supplementary Material

FIGURE S1 **(A,B)** The schematic of the entire pathway networks for MAPK signaling and spliceosome from the KEGG database.

TABLE S1 The PCV2 Cap-interacting cellular proteins in the PCV2 infected (269) and mock infected (87) PK-15 cells, respectively.

TABLE S2 The unique PCV2 infected-interacting host proteins (222).

TABLE S3 The GO annotation analyses of PCV2 Cap-interacting host proteins.

TABLE S4 The KEGG enrichment analyses of PCV2 Cap-interacting host proteins.

TABLE S5 The integrated KEGG pathways and GO annotation analyses of PCV2 Cap-interacting host proteins.

TABLE S6 The interactions network of the confirmed host protein-PCV2 Cap interaction and the cellular partners of the Cap-interacting host proteins.
