# Supplementary material for: Protein Interactions Network of Porcine Circovirus Type 2 Capsid With Host Proteins
Source: Front Microbiol. 2020 Jun 3;11:1129. doi: 10.3389/fmicb.2020.01129 (PMC7283462; doi:10.3389/fmicb.2020.01129)

Supplementary Material

**Supplementary Figure 1.** **Figures 1A, B** The schematic of the entire pathway networks for MAPK signaling and spliceosome from the KEGG database.

Legends of **Figures 1A, B**


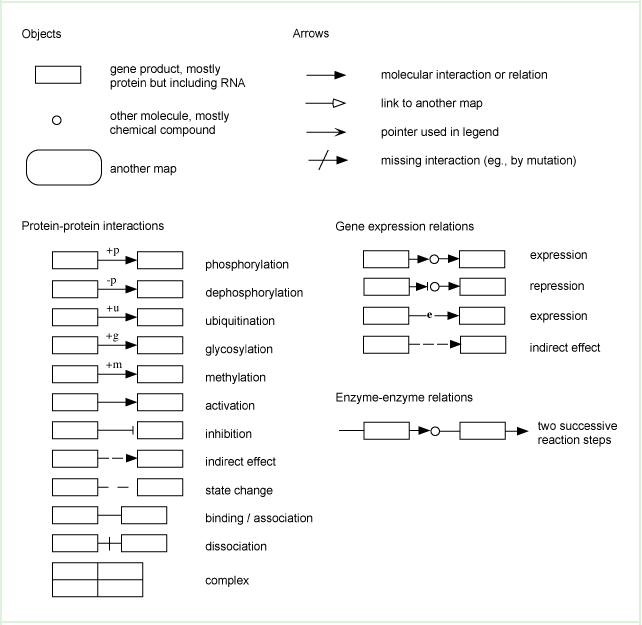


A


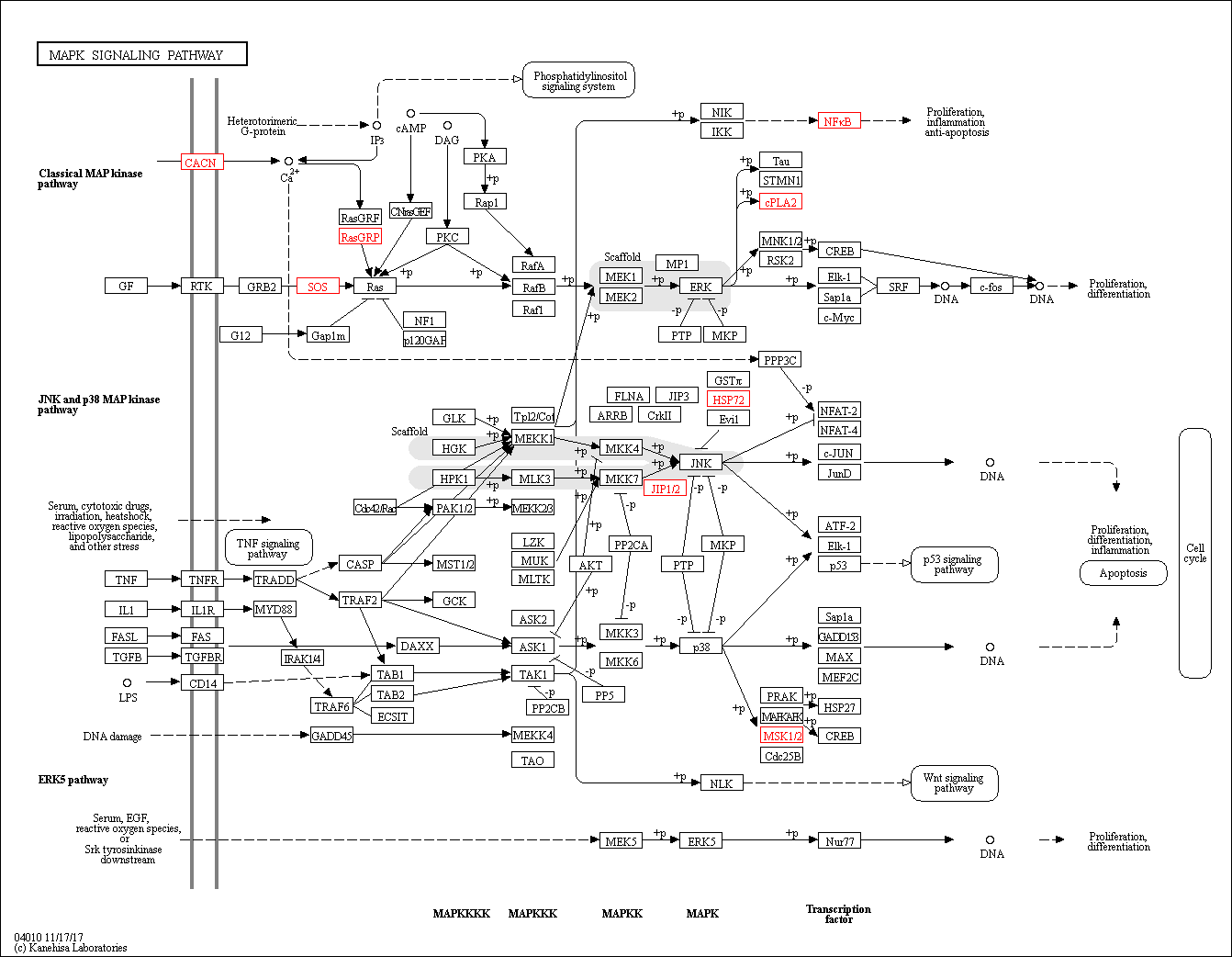


B


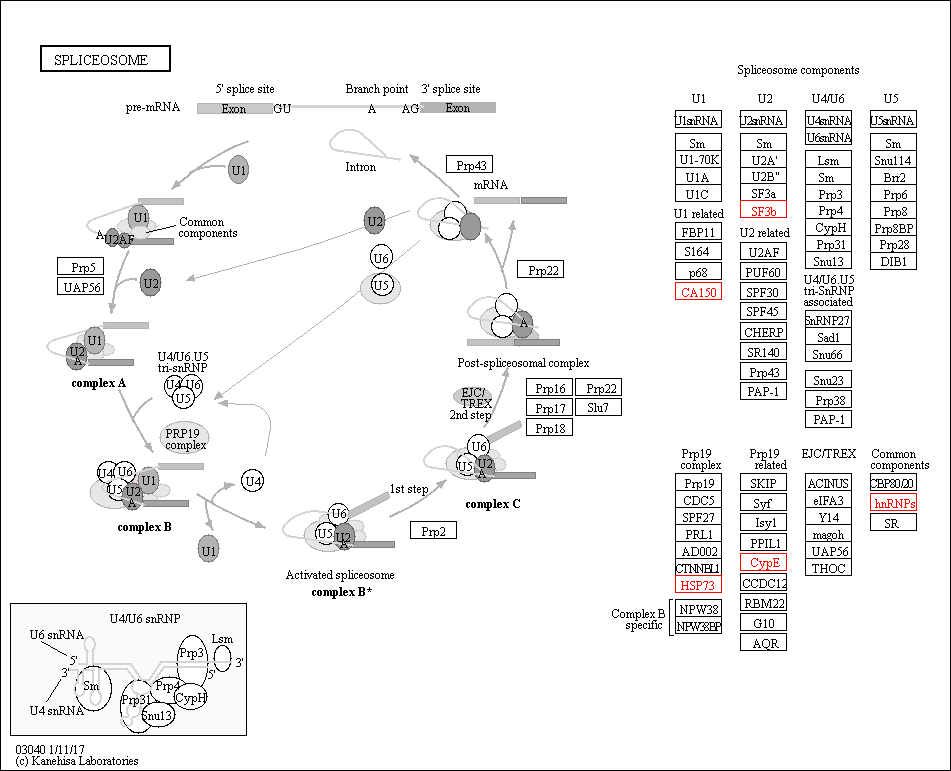

Supplement: FIGURE S1 — (A,B) The schematic of the entire pathway networks for MAPK signaling and spliceosome from the KEGG database. [file Data_Sheet_1.zip › Supplementary Materials.zip/Supplementary Figure 1-The schematic of the entire pathway networks for MAPK signaling and spliceosome from the KEGG database.docx]
